# Hepatitis B virus and celiac disease: HBV and Celiac Disease

**Published:** 2011-01-01

**Authors:** Ceres Concilio Romaldini, Dorina Barbieri

**Affiliations:** 1 Department of Pediatrics, School of Medicine, University of Sao Paulo, Sao Paulo, Brazil

**Keywords:** Hepatitis B Virus, Celiac Disease

Dear Editor,

Regarding the article of Leonardi and La Rosa: "Are hepatitis B virus and celiac disease linked?", published in the 2010 issue of Hepatitis Monthly [1], we believe that there is no relationship between the hepatitis B virus (HBV) and celiac disease (CD). None of the study's patients tested positive for immunoglobulin A anti-endomysium or immunoglobulin A anti-tissue transglutaminase antibodies, nor did they exhibit symptoms of CD. Therefore, there is no reason to confirm a diagnosis of CD. We understand that the interest of the authors to study the possible relationship between CD and HBV is based on their own experience and the evidence of others that untreated patients with CD have a lower percentage of response to HBV vaccine than healthy subjects. However, in celiac children, treatment with a gluten-free diet may improve the immune response to HBV vaccine; the mechanism for this is unclear [2][3]. Several studies have linked both HBV infection and HBV vaccine to a variety of autoimmune manifestations, including autoimmune hepatitis, systemic lupus erythematous, rheumatoid arthritis, polyarteritis nodosa, type 1 diabetes, multiple sclerosis, thyroid disease and uveitis [4]. Although the role of HBV infection in the development of autoimmune diseases has been extensively discussed in the literature, it remains a controversial subject, and variety possible mechanisms have been suggested. Molecular mimicry, based on amino acid similarities shared by viral and self antigens, has long been proposed as a pathogenic mechanism. Iglesias et al. presented two patients who developed CD after resolution of an acute HBV infection. A diagnosis of CD was confirmed by positive serological tests and the presence of the typical histopathologic pattern. These authors suggested that the development of immune response for HBV clearance triggers the intestinal tissue damage observed in CD in genetically predisposed individuals [5]. In addition, a previous study reported activation of CD in susceptible individuals during interferon-alpha therapy for chronic hepatitis C infection. Interferon-alpha has the potential to exacerbate autoimmune disease either by direct effects on tissue or by interacting with the immune system; thus, altering the link between lymphocyte populations and the cytokine production profile [6]. In conclusion, no clinical evidence is available for an association between CD and hepatitis infection, and the occurrence of these two diseases in one patient may simply be a chance finding. The possibility of a cause-and-effect relationship might be better investigated.
